# Circulating Ceramides- Are Origins Important for Sphingolipid Biomarkers and Treatments?

**DOI:** 10.3389/fendo.2021.684448

**Published:** 2021-07-27

**Authors:** Michael Mah, Mark Febbraio, Sarah Turpin-Nolan

**Affiliations:** Cellular and Molecular Metabolism Laboratory, Monash Institute of Pharmaceutical Sciences, Drug Discovery Biology, Monash University, Parkville, VIC, Australia

**Keywords:** ceramides, circulation, biomarkers, cardiovascular, metabolic disease

## Abstract

Biomarkers are important tools for describing the adequacy or inadequacy of biological processes (to allow for the early and accurate diagnosis) and monitoring the biological effects of intervention strategies (to identify and develop optimal dose and treatment strategies). A number of lipid biomarkers are implicated in metabolic disease and the circulating levels of these biomarkers are used in clinical settings to predict and monitor disease severity. There is convincing evidence that specific circulating ceramide species can be used as biological predictors and markers of cardiovascular disease, atherosclerosis and type 2 diabetes mellitus. Here, we review the existing literature that investigated sphingolipids as biomarkers for metabolic disease prediction. What are the advantages and disadvantages? Are circulating ceramides predominantly produced in the liver? Will hepatic sphingolipid inhibitors be able to completely prevent and treat metabolic disease? As sphingolipids are being employed as biomarkers and potential metabolic disease treatments, we explore what is currently known and what still needs to be discovered.

## Introduction

The escalating epidemic of obesity and its metabolic co-morbidities such as type 2 diabetes mellitus (T2DM) and cardiovascular disease (CVD) are major sources of adverse health effects, impaired quality of life and morbidity (1). Despite significant efforts to establish therapies to prevent and treat metabolic disease, the obesity epidemic continues to grow at an alarming rate (1, 2). T2DM is a metabolic condition where the body becomes non-responsive to the actions of insulin and/or cannot produce enough insulin to maintain euglycaemia. T2DM can be managed by diet or physical interventions if diagnosed early, however it usually results in patients requiring medication to maintain their blood glucose and weight. Being overweight or obese is one of the major underlying factors that contributes to T2DM and if left unchecked, T2DM can contribute to a higher risk of CVD events.

CVD refers to a group of diseases that target the heart and blood vessels including coronary, cerebrovascular, peripheral arterial and congenital heart diseases and atherosclerosis. Strokes and heart attacks are considered to be acute events in patients that result from blockages of blood flow to the heart or brain that are predominately associated with increased adiposity, reduced physical activity, hypertension and T2DM. The connections and association between CVD and T2DM have been clearly established with a number of circulating factors being explored as causes, consequences and more recently biomarkers (3, 4). The most recent of these circulating factors is a group of sphingolipids that have been associated with both CVD and T2DM in a number of metabolic disease studies. Elevated serum ceramide and dihydroceramide (precursors of ceramides) species have been found to correlate with CVD and T2DM events or disease severity but also to even predict T2DM onset years before a patient presents to healthcare professionals or a diagnosis has been made (3, 4).

### Sphingolipids

**Figure 1:**
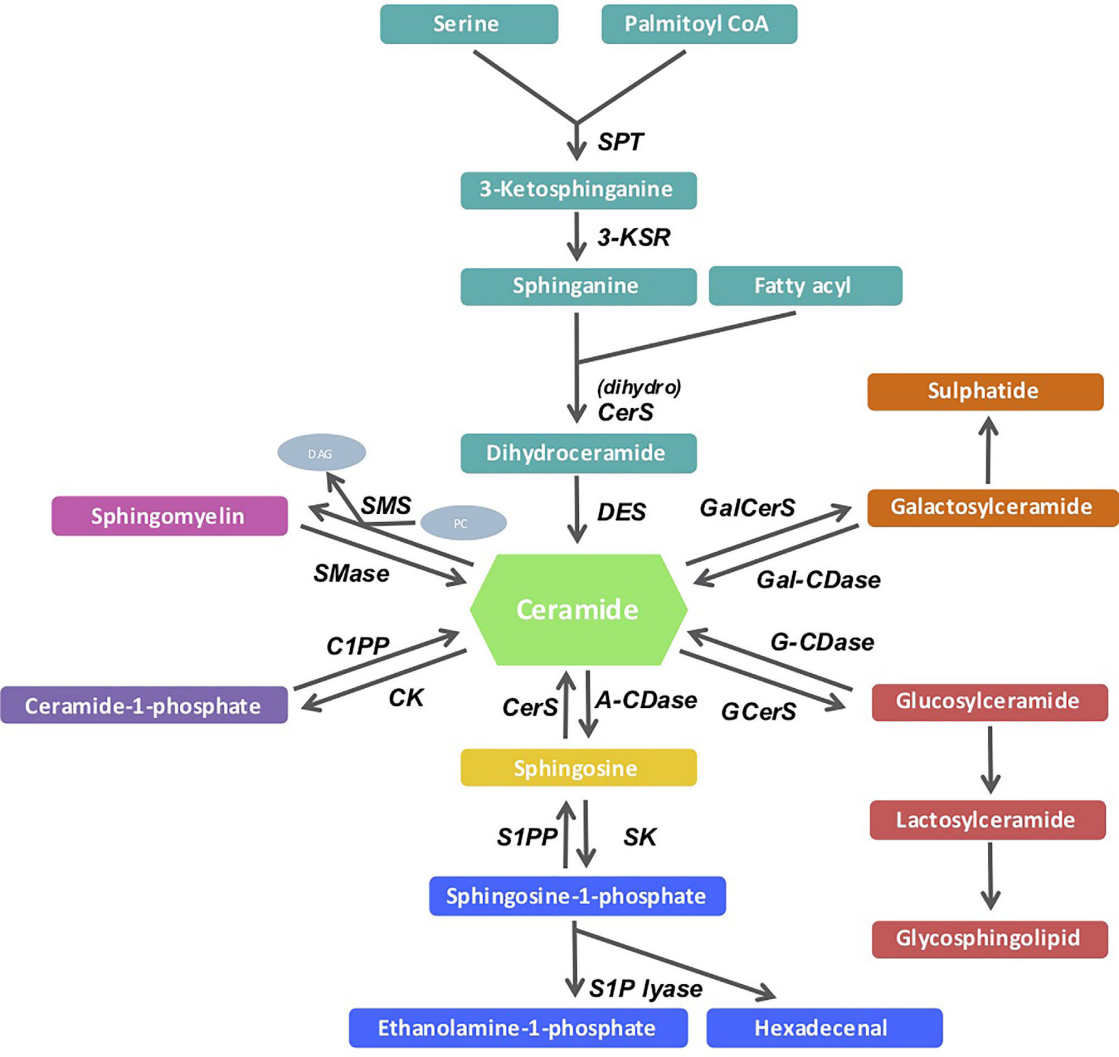
Sphingolipid metabolism pathway. *De novo* (light green arm) sphingolipid biosynthesis begins with the condensation of serine and palmitoyl-CoA, which is catalyzed by the serine palmitoyltransferase enzyme. Its product, 3-ketosphinganine is enzymatically reduced to sphinganine or dihydrosphingosine by 3-ketosphinganine reductase. Sphinganine and fatty acyls then synthesize dihydroceramides under the regulation of the dihydro ceramide synthases (CerS) that are then reduced by dihydroceramide desaturase to produce ceramides of different acyl-chain lengths. Ceramide can also be metabolized *via* other enzymes in the salvage (light blue and pink arms), sphingomyelin hydrolysis (dark green arm) pathways.

Sphingolipid metabolism spans a vast network of biosynthetic pathways and incorporates numerous intermediary products and enzymes. The pathways and disease etiologies have been well documented in recent reviews (5–8). Many of these pathways, converge on the central sphingolipid metabolite known as ceramide (see **Figure 1**). The *de novo* pathway is the most important route of ceramide biosynthesis, and it begins with a condensation reaction between serine and palmitoyl-CoA, facilitated by the enzyme serine palmitoyltransferase (SPT), to form 3-keto-sphinganine. This intermediary product is then reduced by 3-keto-sphinganine reductase to sphinganine. Subsequently, *via* the action of the six mammalian CerS enzymes, sphinganine is acylated to form dihydroceramides of different fatty acyl chain lengths. These transient dihydroceramides are finally desaturated by dihydroceramide desaturase (DES) enzymes, where a 4,5-*trans*-double bond is inserted into the sphingosine backbone to generate distinct ceramide species with different acyl chain lengths and biophysical properties (9, 10). Ceramide has emerged as a crucial mediator of sphingolipid biology, and recent studies demonstrating the regulatory role of these lipids in disease has reinvigorated research efforts in this space (5). While many lipid species are known to accumulate during obesity, ceramide metabolism is of great importance, because it is amongst the most metabolically pathogenic, since it directly interferes with insulin sensitivity (11–13).

Ceramides are bioactive lipid metabolites that increase in metabolic tissues during obesity and induce cellular dysfunction *via* inhibition of the insulin, apoptotic and mitochondrial energy utilisation pathways (14, 15). Ceramide production is regulated by six Ceramide Synthase (CerS) enzymes that each has their own unique fatty acid preference. The addition of the unique fatty acid to a sphingoid backbone, results in the generation of highly specific ceramide species (CerS1 for C_18:0_ ceramides, CerS2 for C_22:0_-C_24:0_, CerS4 for C_18:0_-C_20:0,_ CerS5 and CerS6 for C_16:0_) demonstrating CerSs are paramount for exclusive ceramide manipulation (5, 14, 19–21). This is evident as targeting CerS6 to reduce C_16:0_ ceramides, resulted in the prevention of diet-induced obesity and glucose intolerance (14, 16), whilst the ablation of CerS2 reduced C_22:0_ and C_24:0_ ceramides, increased C_16:0_ ceramides and contributed to spontaneous hepatocellular carcinoma development (18). Additionally, genetic deletion of specific CerSs in animal models exhibiting metabolic dysfunction has been shown to offset high-fat diet (HFD)-induced insulin resistance and impaired glucose tolerance without the negative effects of global ceramide inhibition (13–15, 17, 20, 22–24). By contrast, ablation of other CerS isoforms has led to the development of various abnormalities such as an impaired skin barrier function, myelin sheath defects, hepatocarcinomas, and an increased susceptibility to steatohepatitis (18, 20, 25, 26). These collective efforts have significantly progressed the basic functional understanding of each CerS isoform, but perhaps more importantly, they highlight a need for greater fine-tuning of therapeutics geared towards the CerSs as not all ceramide species confer deleterious effects. The race is on to understand the physiological impact of each distinct ceramide species in key metabolic tissues. This is especially important since it is not always the most predominant ceramide species or CerS enzyme that can have the most detrimental effect.

### Global Ceramide Inhibition

Significant strides have established ceramide signaling as a potent driver of “lipotoxicity” in metabolic diseases. “Lipotoxicity” was a term coined to refer to the deleterious effects that lipids induced *via* intracellular mechanisms. We prefer to use the term “cellular dysfunction” instead as most of the intracellular processes that are altered by ceramides are not toxic but rather negatively influence cellular function. Whilst numerous studies have identified that ceramides contribute to cellular dysfunction (13, 14, 17, 27), there is still no direct evidence, at least in pre-clinical *in vivo* models, that a distinct ceramide species directly contributes to cellular toxicity and/or death. However, systemic inhibition of Asah1 that resulted in the gross accumulation of all ceramide species in various tissues resulted in transgenic mice that were unable to thrive and had a severely shortened lifespan (28). Whilst the direct link between any of the ceramide species and the transgenic animal’s phenotype were not found it does highlight that total ceramide accumulation throughout the body can be detrimental (28).

Distinct ceramide species, or ratios of, can however, be favorable in some tissues (18–20), but not others (14, 20, 23). From a metabolic perspective, the *de novo* synthesis pathway has garnered the most research attention for its major role in ceramide biosynthesis. This is, perhaps, due to the once diet-centric view of ceramide production and how the availability of palmitate and serine may regulate this process (12). While initial studies may have underappreciated the contribution of ceramides to metabolic dysfunction, mainly due to the use of unsuitable lipid cocktails and physiologically disparate cell systems, there is now a clear link between ceramide signaling and defective insulin action (13–15, 20, 22, 23).

While promising, however, pharmacological targeting of ceramide synthesis has been rife with controversy. Myriocin (a *de novo* ceramide synthesis inhibitor) treated mice exhibited a reduced ceramide content in the liver resulting in improved hepatic insulin signaling (13, 29), thereby proposing ceramide synthesis as a novel and promising target for the treatment of obesity-associated insulin resistance. Although ceramide itself is, unquestionably a harmful intermediate, there is a considerable body of evidence to suggest that targeting ceramide metabolism varies greatly depending on the pathway and enzyme of interest, the target tissue, and more recently, the intracellular localization (22). Intensive research into sphingolipid metabolism has revealed that almost forty distinct enzymes act on ceramide as either a substrate or product (30), suggesting that the synthesis of ceramides is very complex. Not only has this rapidly expanded our understanding of the sphingolipid metabolism network, but it has also broadened our view of bioactive lipid species and their contribution to cell biology and disease.

Ceramide biosynthesis encompasses a large network of pathways and enzymatic reactions. Many of these enzymes have garnered interest as potential pharmacological targets, particularly for the treatment of insulin resistance and CVD (13, 14, 16, 29). Early studies attempting to pharmacologically modulate ceramide metabolism have generally focused on enzymatic targets involved in canonical signaling pathways (13, 29). These efforts have yielded great success and have significantly progressed our understanding of the key enzymes involved in sphingolipid metabolism (13–15, 18, 23, 31). It was apparent, however, that global inhibition of ceramide synthesis may, in fact, be detrimental to overall cellular homeostasis (13), however, recent studies have demonstrated that global ceramide manipulation in a controlled manner, i.e., tissue specific or spatially-timed deletion, can indeed be beneficial to treat obese models (23, 24).

## Sphingolipids as Biomarkers of Metabolic and Cardiovascular Diseases

Biomarkers are characteristics of the body that can be easily measured to predict health and disease status. Specific biomarkers are chosen due to their distinctive biological characteristics and must be reliable, reproducible and easy to measure (3). Not only are biomarkers important tools for describing the adequacy or inadequacy of biological processes (to allow for the early and accurate diagnosis), but they also monitor and advise on the biological effects of intervention strategies enabling the identification and development of optimal dosage and treatment strategies (3).

A number of lipid biomarkers are implicated in cardiometabolic disease, and the circulating levels of these biomarkers are used in clinical settings to predict and monitor disease severity. Circulating lipids such as cholesterol, lipoproteins (low, very low or high density) and triacylglycerols (TAGs) are assessed to predict not only metabolic diseases but quite often also for cardiovascular and atherosclerotic diseases. If patients are to have their blood lipids profiled then glucose homeostasis is also assessed by fasting blood glucose levels and haemoglobin A1c. Together the information obtained from these metabolic blood panels is used to predict CVD and T2DM disease severity, future CVD events, or to monitor the effectiveness of current lipid or glucose lowering interventions that are employed to reduce the severity of atherosclerosis, CVD and T2DM. Whilst, collectively, these lipid and glucose homeostasis markers have been successful to assess a patient’s metabolic health, the search continues for more disease specific biomarker rather than multiple biomarker panels that may not conclusively diagnose the patient’s disease or attribute the biomarkers to a specific organ’s dysfunction. The majority of CVD and T2DM biomarkers (lipoproteins, glucose, cholesterol) are produced or regulated by the liver, however we know that the liver is not the sole organ to actively contribute to these diseases. Whilst the production and origin of these metabolic biomarkers is from the liver, other metabolic tissues, such as skeletal muscle, white and brown adipose tissue and pancreas, are actively involved in the clearance of these markers from the bloodstream.

Increased circulating ceramide levels is associated with a variety of metabolic and cardiac pathologies, including obesity, T2DM, insulin resistance and even more convincingly, ceramides could play a role in the onset and pathogenesis of CVD (3, 4, 14, 32–35). There is convincing evidence that specific circulating ceramides can be used as biological predictors and markers of CVD (4, 34, 36), atherosclerosis (4) and T2DM (3, 14, 32–34). However, it is now clear that only certain ceramide species or combinations/ratios of ceramides and dihydroceramides may be responsible for this process. In a recent Frontiers review, Hilvo et al., identified the current literature citing ceramides as biomarkers of CVD and associated cardiac events (35). As this review extensively covered ceramide biomarkers for both CVD and T2DM, we have provided summary Tables of the results discussed (see **Tables 1**, **2**) and focused upon the direct origins of where these ceramide biomarkers are being produced.

**Table 1:** Individual circulating ceramide species.

Disease	C16:0	C18:0	C20:0	C22:0	C24:0	C24:1	C26:0	Disease associations/comments	Ref
**AMI**	↑				↑			Compared to UAP patients	(37)
	↑	↑	↑						(38)
	↑	↑			↑	↑		In STEMI patients: worse in patients with plaque rupture vs. plaque erosion	(39)
	↑	↑			↑	↑		Elevated composite CVD outcome (non-fatal AMI, stroke, CVD death)	(40)
**CAD**		↑		↑		↑			(41)
			↑	↑	↑			LAD stenosis ≥50%, adjusted for CVD risk factors	(42)
	↑	↑				↑		Increased risk of MACE	(4)
	↑		↑			↑		Increased MACE and composite endpoint death or nonfatal ACS	(43)
	↑	↑			↓			In stable CAD patients	(44)
	↑	↑				↑		Predictive of MACE incidence and MACE death	(45)
	↑	↑			↓				(46)
	↑							Increased MACE and composite endpoint death or ACS	(47)
**T1D**			↓	↓	↓		↓	Increased progression to macroalbuminuria	(48)
**T2DM**		↑	↑	↑				Increased BMI and HOMA-IR	(49)
	↑	↑	↑	↑		↑		Positively correlated with insulin resistance	(50)
		↑	↑		↑	↑		Inversely correlated with insulin sensitivity	(51)
	↑	↑						In non-diabetic patients: predictive of insulin resistance, and increased visceral adiposity	(52)
		↑						Positively correlated with BMI, fasting glucose, insulin, and HbA1c; C18:0 strongest predictor of incident T2D	(53)
		↑	↑	↑				Increased susceptibility to develop T2D; ceramides elevated 3 years prior to diagnosis	(3)
	↑	↑	↑	↑				Increased insulin and HOMA-IR	(54)
**Ageing**	↑				↑	↑	↑	Compared to young adults	(55)
		↑	↑			↑		Lower cardiorespiratory fitness	(56)

ACS, acute coronary syndrome; AMI, acute myocardial infarction; BMI, body mass index; CAD, coronary artery disease; CVD, cardiovascular disease; HbA1c, hemoglobin A1c (glycated hemoglobin); HOMA-IR, homeostatic model assessment for insulin resistance; LAD, left anterior descending coronary artery; MACE, major adverse cardiovascular event; STEMI, ST segment elevation myocardial infarction; T1D, type 1 diabetes; T2DM, type 2 diabetes; UAP, unstable angina pectoris; VAT, visceral adipose tissue.

**Table 2:** Circulating ratios of ceramide species.

Disease	$\frac{\text{C}18:0}{\text{C}16:0}$	$\frac{\text{C}16:0}{C24:0}$	$\frac{\text{C}18:0}{C24:0}$	$\frac{\text{C}18:0}{C24:2}$	$\frac{\text{C}20:0}{C24:0}$	$\frac{\text{C}22:0}{C24:0}$	$\frac{\text{C}24:1}{C24:0}$	Disease associations/comments	Ref
**AMI**							↑	More severe coronary stenosis	(37)
		↑						Increased CVD risk	(7)
**CAD**		↑	↑				↑	Increased risk of MACE	(4)
		↑			↑		↑	Increased risk of composite endpoint death or nonfatal ACS	(43)
		↑	↑				↑	Increased risk of CVD death	(44)
			↑				↑	Increased MACE incidence	(45)
		↑				↑	↑	Increased risk of CVD death; C24:0/24:1 (inverse) indicative of reduced CVD death	(46)
		↑			↑		↑	Increased risk of composite endpoint death or ACS	(47)
**T2DM**				↑				Positively correlated with VAT/total-fat ratio, and inversely with lower-body fat/total fat ratio	(52)
	↑							Strongest predictor of incident T2D independent of glucose and HbA1c	(53)
**Ageing**		↑			↑			Compared to young adults	(55)

ACS, acute coronary syndrome; AMI, acute myocardial infarction; BMI, body mass index; CAD, coronary artery disease; CVD, cardiovascular disease; HbA1c, hemoglobin A1c (glycated hemoglobin); HOMA-IR, homeostatic model assessment for insulin resistance; LAD, left anterior descending coronary artery; MACE, major adverse cardiovascular event; STEMI, ST segment elevation myocardial infarction; T1D, type 1 diabetes; T2DM, type 2 diabetes; UAP, unstable angina pectoris; VAT, visceral adipose tissue.

There is no doubt that circulating ceramide species are increased in T2DM and CVD patients. Clinical observations from the Dallas Heart Study, a large-scale multi-ethnic cohort study assessing diabetes outcomes over 7 years, revealed that saturated C_16:0_ and C_18:0_ ceramides in serum positively correlated with insulin resistance as per a homeostatic model assessment of insulin-resistance (HOMA-IR) index (52, 57). What is even more remarkable is that certain circulating dihydroceramide and ceramide species in both mice and humans were able to predict metabolic disease onset up to 9 years before T2DM events began to occur (3).

Circulating ceramide levels have also been suggested to predict the risk of cardiovascular events more accurately than traditional risk factors of LDL-cholesterol or HDL-cholesterol (44, 58). The therapeutic potential and benefit of using circulating ceramides as predictors of CVD also appears to be consistent across ethnicity (40, 52, 57, 59).

Whilst the increases of individual ceramides species, such as C_16:0_, C_18:0_ and C_24:0_, are the predominate species that are elevated in the majority T2DM and CVD patient studies (**Table 1**), it is obvious that increases in these specific ceramides are not isolated to one specific CVD or T2DM disease. As identified in **Table 1**, multiple studies have identified a number of species which are modulated by their respective metabolic or cardiovascular diseases. This raises the question, are single ceramide species ideal biomarkers if they cannot distinguish between different cardiac events or even CVD and T2DM? An alternative to single ceramide specie biomarkers has been addressed by the Mayo Clinic, who suggest that ceramide predictive scores could be a more sophisticated biomarker tool by incorporating ceramide species ratios (35). **Table 2** indicates that different ceramide ratios do appear to be distinctly different in T2DM patients compared with CVD patients. Due to the low number of T2DM studies evaluating the ceramide ratios, only time will tell if this holds true with increased studies. These studies collectively have revealed a novel and exciting avenue, not only for understanding how ceramides function in the body, but that circulating ceramides can also be a useful diagnostic tool for T2DM and CVD. Perhaps the stigma of ceramides being “toxic” will now begin to fade. As the usefulness and potential of circulating ceramide species as biomarkers of metabolic diseases continue to emerge, it is clear that we still lack the knowledge of which tissues these ceramide species are derived from and how they are transported around the body.

## Origins of Ceramides

**Figure 2:**
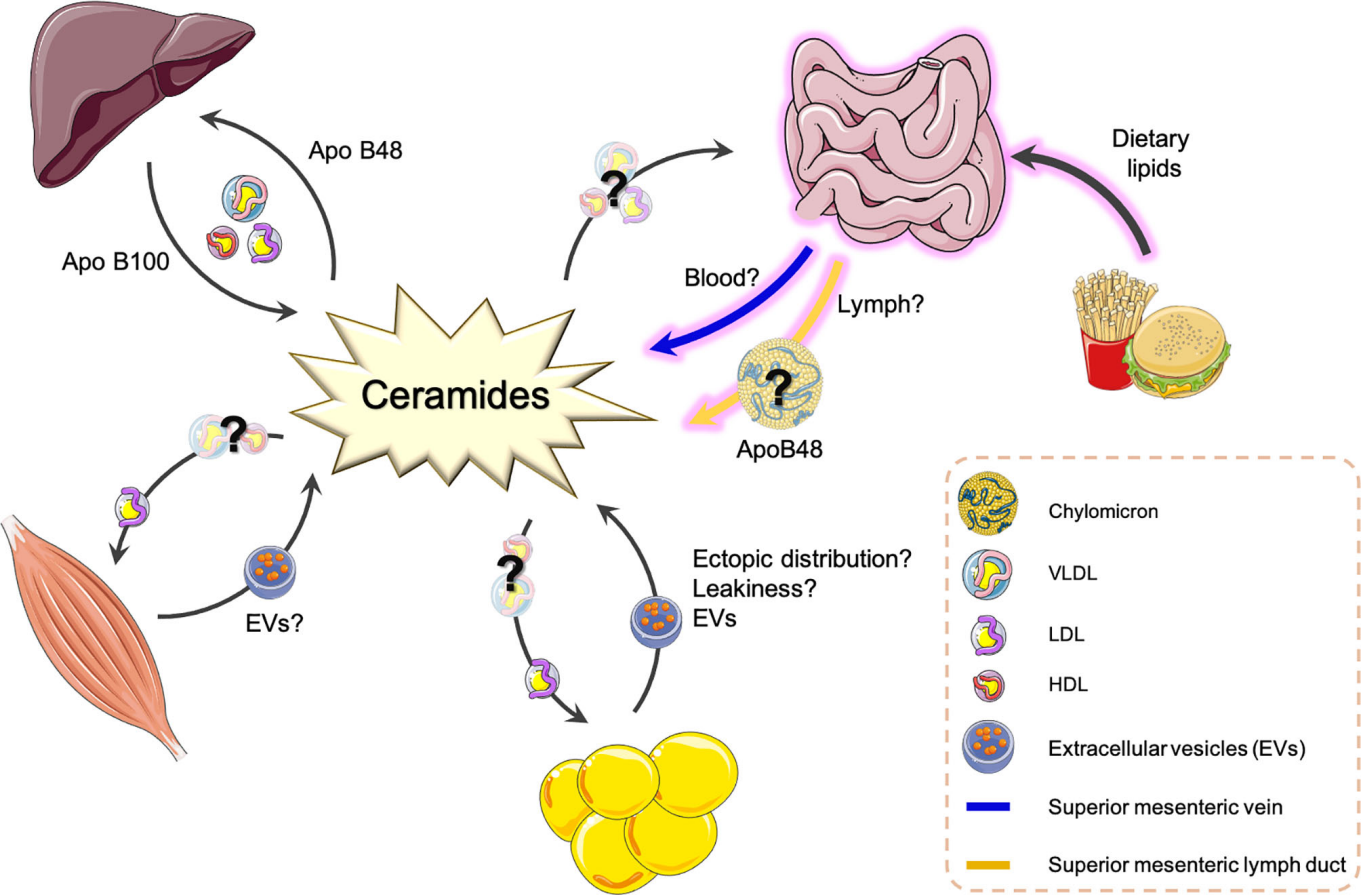
Potential origins and transport routes of ceramide species around the body. This is a summary of known transport routes and packaging systems of ceramide species as determined from the current literature. Question marks and broken line arrows are suggestive of new routes and transport routes mechanisms that have recently been proposed or published. ApoB, Apolipoprotein protein; EV, Extracellular vesicle; LDL, low density lipoprotein; VLDL, very low-density lipoprotein; HDL, high density lipoprotein.

Ceramide species content and ratios are fast becoming the most predictive biomarkers for T2DM and CVD disease appearance and progression. It is insufficient, however, to simply measure these sphingolipids without understanding the biology of their production or organ sites of production. Over the past 20 years, research has identified how sphingolipids are transported within basic cell systems, however, we still lack the knowledge of the transport mechanisms that exist from cell to cell and organ to organ within the body and how this adapts during metabolic disease. To complicate things even further, we still lack the basic physiology of how multiple metabolic organs, together, contribute to T2DM and CVD and their associated metabolic diseases. Knowing where these circulating sphingolipid biomarkers are originating from and being directed to can only enhance more specifically targeted pharmacological inhibitors to treat T2DM and CVD and their associated pathologies. The following sections will investigate what is currently understood in the packaging and export of specific ceramide species and explore new transport avenues that could be manipulated in the future (**Figure 2**).

### Liver

The liver has been the most widely studied organ with respect to lipid uptake, metabolism and export. It is well understood that the liver is one of the most active users and producers of lipids in the body. The liver not only takes up lipids for energy utilization *via* beta-oxidation but can repackage vast amounts of different lipids to either be stored within the cell or repackaged with lipoproteins for export back into the circulation. Furthermore, the liver is a major site of excess nutrient conversion, where surplus carbohydrates and proteins are readily metabolized into lipid moieties and incorporated into newly synthesized lipoproteins (VLDL), cholesterol or phospholipids. Other lipoproteins such as LDL and HDL are derived from hepatic VLDL but are hydrolyzed within the circulation to generate LDL and HDL.

More recently, the liver has been demonstrated to play major roles in metabolic disease pathologies such as obesity and T2DM when distinct ceramide species are manipulated by genetic animal models (13, 14, 17, 20, 22, 60). Mice lacking functional CerS6 were refractory to the detrimental effects of HFD resulting in marked improvements in glucose handling, attenuated pro-inflammatory disposition and increased rates and efficiency of hepatic lipid metabolism (14). Furthermore, it was determined that the improvements in lipid metabolism of both global and hepatic CerS6 knockout animals (14, 22) was due to C_16:0_ ceramides interacting with mitochondrial fission factor to promote hepatic mitochondrial fragmentation (22).

*De novo* synthesis enzymes DES1 and SPT, have also emerged as potential targets for ceramide reduction strategies. Liver-specific or whole-body excision of DES1 was shown to resolve insulin resistance and hepatic steatosis in both *Lep^ob/ob^* mice and HFD-fed animals (23). The proportion of dihydroceramides also drastically increased in mice lacking *DES1*, and this was evident in the liver, white adipose tissue, soleus muscle, and serum (23). Interestingly, very long chain ceramides (C_22:0_-C_24:0_) were depleted in these animals, which was surprising as these specific ceramides have previously been associated with a more favorable metabolic phenotype (52). Therefore, it remains to be seen how DES1 inhibition may translate to insulin resistance and hepatic steatosis in a clinical setting, of which underlies many major cardiometabolic complications.

Genetic ablation of Sptlc2, a catalytic subunit of the SPT enzyme, significantly alters the sphingolipid profile in transgenic animals, particularly ceramide and sphingomyelin subspecies (40). Liver-specific deletion of Sptlc2 has a dramatic effect on sphingolipid levels in circulation. Ceramides and sphingomyelins, in particular, were significantly reduced in plasma from liver-specific Spltc2-deficient animals (61). This translated to an overt anti-atherogenic phenotype as determined by elevations in plasma ApoE, but not ApoB or ApoA-I, and an enhanced cholesterol efflux efficiency from macrophages (61). Interestingly, however, conventional Sptlc2 knockout mice have been shown to develop hepatic tumors (59), as well as necrotic lesions in gastrointestinal cells lining the small and large intestine (60).

Additionally, CerS5-derived C_16:0_ ceramides have also been identified to negatively impact insulin sensitivity and glucose tolerance in HFD challenged *CerS5* deficient mice (17). Gosejacob et al., found significant reductions of C_16:0_ ceramides in the liver, however unlike the studies from the Brüning laboratory (14, 22), the reduction of the hepatic ceramide species surprisingly did not result in lowered circulating C_16:0_ ceramide levels (17). Furthermore, the metabolic phenotype observed in the CerS5 global knockout by Gosejacob et al., are in direct contrast to those from Hammerschmidt et al., where it was reported that CerS6, but not CerS5, was necessary for obesity-driven insulin resistance and mitochondrial dysfunction (17, 22). Unlike CerS6-knockout animals, deletion of the CerS5 gene by Hammerschmidtt et al., failed to protect against obesity-induced hepatic lipid accumulation and impaired glucose tolerance (22).

CerS2-derived very long chain ceramides also have important metabolic implications in the liver. Previous studies suggest CerS2-derived C_22:0_ and C_24:0_ ceramides may not be deleterious in liver, as knockdown of CerS2 led to glucose intolerance, impaired insulin signaling in the liver and the development of hepatic tumors in mice (18, 62). In terms of the changes to the hepatic sphingolipid profile, a loss of the CerS2 gene, as expected, completely abolished synthesis of C_22:0_ and C_24:0_ ceramides but led to an unexpected increase in levels of C_16:0_ ceramide (19, 25). Similar maladaptive outcomes were observed in obese CerS2 heterozygous mice (CerS2^-/+^) (20). Haploinsufficiency of CerS2 rendered mice susceptible to hepatic steatosis and slight impairments in glucose tolerance and insulin resistance potentially due to a clear shift in the ceramide acylation pattern, as C_16:0_ ceramides dramatically increased to compensate the C_24:0_ ceramide depletion (20). The compositional shift in sphingolipid content was also reflected at the transcriptional level, where CerS2 expression in heterozygous animals fell to half that of its corresponding control and CerS6 was slightly upregulated (20).

There is evidence to show that hepatic-derived ceramides are packaged into lipoprotein particles derived from the liver and transported to peripheral tissues (24, 63, 64). The first study to identify lipoprotein-containing hepatic-derived ceramides was conducted in rat hepatocytes incubated with radiolabeled ceramide and sphingomyelin. While most of the radiolabeled sphingolipid substrate was associated with the internal hepatocellular compartment, a small proportion of substrate was secreted into the culture medium as VLDL-containing ceramides (63). Furthermore, lipoproteins isolated from rat plasma also contained ceramides, thus identifying that hepatic-derived ceramides could be exported into the circulation with VLDL particles which contained significantly greater ceramide levels than LDL or HDL (63). In agreement, liver biopsies from human patients with suspected NAFLD were also shown to contain dihydroceramide species within VLDL particles (65). Plasma dihydroceramide content was also assessed and positively correlated with hepatic steatosis and steatohepatitis indices (65). Further, a separate study identified that human plasma ceramides are predominantly transported within VLDL and LDL particles (66).

The concept of hepatic-derived ceramide export *via* VLDL has also been confirmed in a transgenic mouse line overexpressing acid ceramidase (a catabolic enzyme that facilitates ceramide hydrolysis) (24). In this study, the rates of hepatic VLDL export were increased in animals selectively overexpressing hepatic ceramidase, however these animals also had reduced hepatic lipid content and TAG clearance which resulted in elevated serum TAG levels (24). As ceramidase initiates ceramide hydrolysis to release sphingosine and free fatty acids, the resulting influx of serum TAGs from a constitutively active hepatic ceramidase was of concern.

These collective findings highlight an important axis through which ceramides can be packaged and trafficked from the liver and has potential therapeutic appeal to not only impact hepatic lipid metabolism to improve glucose homeostasis, but to also reduce the appearance of ceramide species in the circulation to prevent their uptake and accumulation in other metabolic tissues. It is no surprise that multiple pharmaceutical companies are currently incorporating hepatic sphingolipid inhibitors into their metabolic research and development programs. Although ceramides have been discovered in various lipoprotein fractions, the liver is not, however, the only organ to package and export ceramides into the circulatory system. It is one of the last organs to receive circulating lipids after a meal. This then begs the question, is it the best target for manipulating ceramide levels to improve metabolic dysfunction?

### Skeletal Muscle

The accumulation of ceramides in skeletal muscle has been extensively studied for the past 30 years with numerous reviews demonstrating how skeletal muscle ceramides are synonymous with defective insulin action (5, 7, 8, 67, 68). As the years have passed, new research findings have attempted to identify the specific insulin signaling targets (11, 12) and more recently, the ceramide species responsible (15, 33, 69). In skeletal muscle, the predominant CerS isoform, CerS1, has now been identified as a negative regulator of insulin sensitivity (15, 33). Studies in CerS1-global and muscle specific knockout mice revealed marked improvements in glucose tolerance and systemic insulin sensitivity (15). Conversely, a potent isoform selective CerS1 antagonist P053, selectively inhibited CerS1 and reduced skeletal muscle C_18:0_ ceramides in by ~50% in obese mice, however despite reductions in whole-body fat mass and a heightened capacity for fatty acid oxidation and mitochondrial respiratory chain function, there was no improvement in glucose metabolism (69). These results were more in line with the global CerS1 knockout mouse that was protected against weight gain, adiposity, and impaired energy expenditure (15, 69). One interesting observation resulting from the study by Turpin-Nolan et al., was that despite a ~90% reduction of C_18:0_ ceramides in skeletal muscle, this did not appear to change circulating C_18:0_ ceramide levels indirectly suggesting that the skeletal muscle does not produce and export ceramide into the circulation as alluded to by Tonks et al. (15, 33). Indeed, further studies are warranted to confirm this before conclusions can be drawn as to the contribution of skeletal muscle-derived ceramides to the circulation, but it appears that skeletal muscle is more important in the uptake of lipoproteins rather than the export of them. However, this is not to say that the skeletal muscle is not able to package and release ceramides at all.

A new field of research investigating the production and release of extracellular vesicles (EVs) has identified that sphingolipids and ceramides can be contained within, and secreted by, skeletal muscle derived EVs (70). Both skeletal and cardiac muscle have been identified to package and release EVs in response to “stress” triggers like muscle contraction or myocardial ischemia (70, 71). Could muscle EVs be a transport mechanism for the release of ceramides and other sphingolipids into the circulation?

### Adipose Tissues

Adipose tissue is commonly thought of as the lipid storage site in the body. Adipocytes swell as they store excess lipid-derived energy and hold onto it until systemic signals arrive to burn it, release it, or increase the secretion of adipocyte factors such as lipids, peptide, cytokines and adipokines to enable the metabolic regulation of other tissues. Ceramide levels have been consistently demonstrated to increase in adipocytes during whole body nutrient excess and contribute to adipocyte metabolic dysfunction (5, 6, 67, 68). Numerous reviews have expertly reviewed how different ceramides and animal models contribute to adipocyte and whole-body insulin resistance and we refer readers to those for a mechanistic insight into this dysfunction (6, 67). Different ceramide species are now being pursued as potential therapeutic targets for insulin resistance and other metabolic abnormalities. Two independent studies have identified ceramide content in adipose tissues of insulin resistant patients are increased (72, 73). Whilst Kolak et al. (72) demonstrated adipose tissue C_24:1_ ceramide species were increased in insulin-resistant individuals, Chaurasia et al. (73) identified C_16:0_ ceramides not C_24:1_ ceramides to be increased in weight matched obese diabetic patients. Previously, Turpin et al. had also demonstrated that human white adipose tissue expression of CerS6, responsible for the production of C_16:0_ ceramides, strongly correlated with key metabolic indices such as insulin resistance, BMI, and whole-body fat mass (14). Clearly increased ceramide accumulation in the adipose tissue contributes to whole body metabolic dysfunction but is this solely from intrinsic mechanisms within the adipocytes or due to increased transport of ceramides to and from adipocytes?

Like skeletal muscle, adipocytes have also been recently discovered to secrete lipid-containing exosomes and EVs (74–77). Flaherty et al., isolated murine adipocyte-derived exosomes and performed lipidomic analysis (74). This identified the lipid classes present in adipocyte-derived exosomes extracted from the circulation of lean and *Lep^ob/ob^* mice. Sphingomyelin and TAGs both represented ~8% of the total lipid content, whilst phosphatidylcholine and free cholesterol were the most abundant with ~25-28% each. Interestingly, ceramide was detected to contribute ~4% of the overall adipocyte-derived exosome’s lipid content, whilst diacylglycerol contributed >2% of the exosome’s lipid content (74). This is one of the first studies to identify that adipocytes can package and release sphingolipids and ceramides into the circulation. Unfortunately, there was no difference in total ceramide levels between lean and obese (Lep *ob/ob*) perigonadal adipose-derived exosomes (74). Crewe et al., were able to isolate small EVs from subcutaneous adipose depots and determined that these particular EVs contained all ceramide species (77). Unfortunately, the ceramide species presented by Crewe et al., are corrected to the ceramide specie content in adipose endothelial cells which does not tell us which ceramide species are the most abundant within the adipose-derived EV samples (77). Additionally, studies must confirm that these adipose-derived EVs are indeed entering the circulation for transport as the EVs were isolated from adipose tissues not the circulation. Experiments determining which sphingolipid and ceramide species are being packaged into these exosomes would be most exciting, especially in combination with other adipose tissue depots to identify if there are specific ceramide species being packaged and transported by subcutaneous versus gonadal adipose depots. It is going to be exciting to follow future publications that will be able to detail where these adipocyte-derived exosomes and EVs are travelling to in the circulation.

### Gut and Lymphatics

The role of the gut as a mediator of nutrient and metabolic homeostasis has been recognized as increasingly important over the past 20 years (78). While the gut microbiome has received the most attention in how the gut regulates metabolic homeostasis, excess nutrient uptake by the intestines can also contribute to the development of metabolic disease (79, 80). The intestinal-lymphatic system is well characterized for the distribution of TAGs and other macromolecules to the systemic circulation but has been severely overlooked as a conduit for ceramide transport throughout the body. Whilst numerous reports have shown that ablating key enzymes in TAG pathways within the intestine can significantly improve lipid metabolism, it has not been without consequences (81–83) and to date there are no promising therapeutic targets.

It has become increasingly clear that the intestinal-lymphatics system has a diverse role in metabolic disease progression (84). Yet, our understanding of its lipid transport function, pertaining to ceramides, is rudimentary. Few studies have identified the presence of different ceramide species in lymph (85–87), but are still unsure of the packaging and transport mechanisms that might regulate intestinal ceramide release.

Chylomicrons are the main lipoprotein synthesized in the small intestine and are responsible for transporting dietary fats from the intestine to the general circulation *via* the lymphatic transport system (88). Of the four main classes of lipoproteins, chylomicrons are by far the largest and the most TAG-rich particles bundled with trace amounts of protein, vitamins, and cholesterol. A unique structural component of intestinal-derived chylomicrons is apolipoprotein (Apo) B48, a hydrophobic, nonexchangeable apolipoprotein (88). ApoB48-containing chylomicrons are exclusively synthesized in the small intestine and serve as key regulators of intestinal lipid uptake and transport. Unlike ApoB100 chylomicrons derived from the liver, ApoB48 chylomicrons are more efficiently catabolized in circulation and is another circulating biomarker for CVD risk (89, 90). Thus, highlighting that the gastrointestinal release of lipids and proteins into the circulation *via* the lymphatics could be the biomarker’s origin that is informing us about each patient’s cardiovascular and metabolic disease prediction and severity.

The *de novo* biosynthesis of ceramides in intestinal epithelial cells (IECs) is predicted to occur in a similar fashion to other metabolic cell types such as hepatocytes, myotubes and adipocytes, however as studies in intestinal ceramide metabolism are lacking this remains to be confirmed. In terms of ceramide export and transport from IECs to other key metabolic sites such as the liver, there is a considerable knowledge gap. The intracellular fusing of lipid-poor ApoB-particles with TAG-rich particles, occurs in the Endoplasmic reticulum, the most important organelle for ceramide synthesis, and continues through to the Golgi before release. While ceramides have been detected in VLDL, LDL and HDL particles in human serum (64), these are not the major intestinal lipoproteins. To date, only a handful of studies have identified the existence of ceramide species in lymph (85–87). These studies, while not designed to uncover a specific transport mechanism, have assessed the lymphatic intestinal absorption of dietary sphingolipids such as glucosylceramide and sphingomyelin. However, in each of these studies, lymph was collected from the thoracic duct which receives drainage from other peripheral sites such as the liver (91). This may have inadvertently led to an overestimation of lipid absorption from the intestinal region as it is impossible to distinguish the mesenteric contribution from this lymphatic sample site.

As TAG inhibition within the intestinal epithelium has proven non-viable, targeting specific ceramide species may be a more attractive lipid target. Within the ceramide space, the role of these bioactive lipids in the intestinal epithelium is completely unknown. Only a handful of studies have measured ceramide levels in the small intestine, but these efforts have not provided a mechanistic link to metabolic disease. One paper investigating the role of the intestinal farnesoid X receptor (FXR) in hepatic glucose metabolism has alluded to this signaling mechanism (92). Lipidomic analysis of serum and ileal tissue harvested from HFD-fed mice lacking the intestinal FXR revealed marginal decreases in multiple ceramide levels (C_16:0_-C_24:0_), which coincided with a substantial inhibition of hepatic gluconeogenesis (92). In this study, suppression of ceramide species in the intestine presumably translated to improvements in the liver. However, it is unclear whether intestinal-derived ceramides are transported directly to the liver, or if they are a major contributor to systemic levels as the liver is currently viewed as the predominant source of circulating ceramides (14, 64). Therefore, identification of the mechanism that underlies ceramide trafficking through the intestinal-lymphatic axis into the circulation will be important for future therapeutic programs looking to target whole-body ceramide dissemination.

## Conclusions

The emergence of circulating ceramides as biomarkers to predict and diagnose CVD and/or T2DM has brought attention to their importance. Once considered “toxic” they now appear to be rebranded as useful informants and important tools enabling disease detection. Furthermore, circulating sphingolipid biomarkers are helping to monitor therapeutic interventions which may or may not directly, or even indirectly, target ceramide production. While still in their biomarker infancy, it is becoming increasingly apparent that ceramide species biomarkers hold true across different metabolic diseases (CVD and T2DM), and importantly, they appear to be unaltered by ethnicity. However, the question now arises, as there are multiple individual ceramide species and ratios being put forward as the best markers for each individual disease that contributes to CVD or T2DM, is there a singular best option or will patient results remain open for interpretation? Can CVD and T2DM be evaluated independently and efficiently if they are being predicted by the same ceramide species ratios? These are the new concerns that need to be considered. Perhaps understanding the origins and target tissues of these circulating ceramides might help to inform which individual species and/or ratio might be the most instructive for the patients’ disease diagnosis. Future studies, animal and human, can only help to guide these new biomarkers and ceramide targeting therapeutics in the right direction.

## Author Contributions

ST-N was the primary author who was responsible for the design and writing of the manuscript. MM and MF assisted with writing and editing the manuscript. All authors contributed to the article and approved the submitted version.

## Conflict of Interest

The authors declare that the research was conducted in the absence of any commercial or financial relationships that could be construed as a potential conflict of interest.

## Publisher’s Note

All claims expressed in this article are solely those of the authors and do not necessarily represent those of their affiliated organizations, or those of the publisher, the editors and the reviewers. Any product that may be evaluated in this article, or claim that may be made by its manufacturer, is not guaranteed or endorsed by the publisher.
